# Knowledge, attitude and practice of invasive mechanical ventilation protocolized weaning among ICU nurses and its influencing factors: A cross-sectional study

**DOI:** 10.1371/journal.pone.0343839

**Published:** 2026-03-06

**Authors:** Li Wang, Yating Yu, Yan Zhu, Qin Zhang, Lei Sun, Juan Chen, Li Xiao, Ping Jia

**Affiliations:** 1 Department of NICU, Sichuan Provincial People’s Hospital, University of Electronic Science and Technology of China, Chengdu, China; 2 Department of PICU, Sichuan Provincial People’s Hospital, University of Electronic Science and Technology of China, Chengdu, China; 3 Department of Hepatobiliary Surgery, Neijiang First People’s Hospital, Neijiang, China; 4 Day surgry ward, Sichuan Provincial People’s Hospital, University of Electronic Science and Technology of China, Chengdu, China; 5 University of Electronic Science and Technology of China, Chengdu, Sichuan Province, China; 6 Department of ICU, Sichuan Provincial People’s Hospital, University of Electronic Science and Technology of China, Chengdu, China; 7 Department of Geriatric cardiovascular ward, Sichuan Provincial People’s Hospital, University of Electronic Science and Technology of China, Chengdu, China; Florida International University, UNITED STATES OF AMERICA

## Abstract

**Background:**

Many foreign countries have implemented protocolized weaning led by respiratory therapists (RTs) or nurses, which can reduce the duration of mechanical ventilation. At present, the implementation of ventilator weaning in China is led mainly by doctors.

**Objective:**

To investigate the status quo of the knowledge, attitudes and practices of adult intensive care unit (ICU) nurses in tertiary hospitals concerning invasive mechanical ventilation (IMV) protocolized weaning and to analyze its influencing factors.

**Methods:**

This cross-sectional study was conducted from September to November 2023 in 30 tertiary hospitals in Sichuan, Chongqing, and Nanjing, and included a total of 700 nurses. Data were collected using a questionnaire designed by the research team. The questionnaire consisted of 39 items, including 3 dimensions: knowledge (concepts and precautions of protocolized weaning, weaning screening and spontaneous breathing trial (SBT) evaluation, SBT methods, extubation knowledge), attitude (professional perceptions, personal beliefs, learning willingness) and practice (implementation of weaning screening assessment and SBT, implementation of extubation). Data analysis was performed using IBM SPSS Statistics, version 25. Univariate analysis was performed using a t test or analysis of variance, while multifactor analysis was conducted using multiple linear regression analysis.

**Results:**

A total of 700 questionnaires were recovered, and after the exclusion of invalid questionnaires, 643 valid responses remained. Among the 643 nurses, 65.47% were from the general intensive care unit (GICU), 86.00% of nurses’ ICU weaning were led by doctors, 43.08% of nurses did not participate in the knowledge of weaning training after work, and 94.56% expressed their willingness to participate in the training of protocolized weaning. The total standardized score of the knowledge, attitudes and practices related to the protocolized weaning of ICU nurses was 82.70 ± 10.69 points, and the standard scores for the dimensions of knowledge, attitudes, and practices were 78.50 ± 16.67, 87.96 ± 10.76, and 76.53 ± 15.51, respectively. Pearson correlation analysis revealed that the correlation coefficients between knowledge and attitude, knowledge and practice, and attitude and practice were 0.274, 0.325, and 0.491, respectively, which were significantly positive correlations (*P* < 0.01). The employment form, whether the ICU has a protocolized weaning program, whether the nurse is willing to participate in the protocolized weaning training, ICU type, and the position were the main factors influencing the ICU nurses’ IMV-protocolized weaning knowledge scores (*P* < 0.05). The professional title, ICU type, whether the ICU has a protocolized weaning program, the role of nurses in weaning decision-making, and whether the nurse is willing to participate in the protocolized weaning training were the main factors influencing ICU nurses’ IMV protocolized weaning attitude score (*P* < 0.05). Gender, ICU type, the position, whether the ICU has a protocolized weaning program, the role of nurses in weaning decision-making, the number of weaning training times in the past 3 years, and whether the nurse is willing to participate in the protocolized weaning training, were the main factors influencing ICU nurses’ IMV-protocolized weaning practice score (*P* < 0.05).

**Conclusion:**

The study found that the participants’ knowledge and practice of the protocolized weaning from IMV were at a moderate level, but they had a more positive attitude. Most participants were willing to participate in the protocolized weaning training. The development of a protocolized weaning program and the provision of related training can promote nurses’ understanding of weaning and increase their willingness to participate in weaning.

## Introduction

Invasive mechanical ventilation (IMV) with an endotracheal tube (ETT) is the basic method to open the respiratory tract for emergencies, major surgeries and critically ill patients and is an important means of life support [1]. Approximately 40% of patients in the intensive care unit (ICU) require IMV treatment [2], and with an increase in an aging population, an increasing number of people need IMV treatment [3]. However, long-term mechanical ventilation (MV) can lead to more complications [4], such as ventilator-associated pneumonia (VAP) [5], ventilator-induced diaphragm dysfunction (VIDD) [6], ICU-acquired weakness (ICU-AW) [7], laryngeal edema [8], and delirium [9], all of which increase the length of hospital stay, medical costs [10] and mortality [5].

Protocolized weaning [11–13] includes three main steps: weaning screening evaluation, a spontaneous breathing trial (SBT) and tracheal extubation, with certain standards for each step. Protocolized weaning can promote early weaning and reduce patient MV time and ICU length of stay [12]. The American Weaning Guidelines [14] recommend that adult patients with MV > 24 h should be weaned with a protocolized weaning scheme. Maria P et al.[15] called for the implementation of protocolized weaning in low- and middle-income countries, which not only improved the clinical outcomes of patients with MV in the ICU but also reduced medical costs.

In 2001, the United States issued “Mechanical ventilator weaning protocols driven by nonphysician health-care professionals: evidence-based clinical practice guidelines” [16], which recommended that nonphysician health care professionals be included in the development and utilization of protocolized weaning protocols (not limited to weaning). Many countries have subsequently explored and implemented nurse-led protocolized weaning with good results [17,18]. ICU nurses play a vital role in promoting good interprofessional cooperation, managing team interactions and driving the weaning process [19]. A systematic review [11] revealed that protocolized weaning led by nurses can reduce the MV time and ICU stay of patients, which is important for patients. In North America, mainly respiratory therapists (RTs) lead the implementation of protocolized weaning, while in Europe, Australia and New Zealand, where weaning is coordinated jointly by doctors and nurses, ICU nurses play the role of RTs and have high autonomy in weaning decision-making [20]. Malaysian scholars have designed a nurse-led clinical practice guide for ventilator weaning nursing training [21] to promote the decision-making ability of nurses’ in clinical weaning; an advanced nursing education course on MV weaning was also developed to better cultivate the judgment and decision-making ability of nurses’ clinical weaning [22]. A shortage of RTs exists in China; 43.9% of RTs are nurses who transitioned to RT through 6 months of on-the-job training [23]. Domestic ICU nurses play an irreplaceable role in the process of IMV weaning. Nurse-led protocolized weaning is still in its infancy in China [24]. A recent study [25] revealed that compared with physician-led empirical weaning, nurse-led protocolized weaning can increase the success rate of weaning, shorten the weaning time, and reduce the MV time and ICU length of stay. To a certain extent, these findings showed that China can also explore the implementation of nurse-led protocolized weaning. Weaning education for nurses can shorten the MV time of patients [26]. Nurses’ participation in ventilator management depends on appropriate knowledge and skills [27]. If they do not have sufficient knowledge and skills, they cannot correctly implement the weaning procedure, which can have serious consequences for patients. Therefore, this study aims to provide a preliminary understanding of the current status and influencing factors of knowledge-attitude-practice among ICU nurses on IMV protocolized weaning in China to provide some reference information for optimizing medical collaboration strategies, improving nurses’ understanding of the role of weaning, implementing nurse-led protocolized weaning and improving the management of the subsequent ventilator weaning process.

## Materials and methods

### Study design

This study is an analytical cross-sectional study aimed at preliminarily understanding the knowledge, attitudes, and practices status and influencing factors of adult ICU nurses in tertiary hospitals in China on IMV-protocolized weaning.

### Sample and setting

The sample size was calculated according to the statistical formula [28] $n=\frac{U_{\alpha /2}^{\text{2}}\sigma^{\text{2}}}{\delta^{\text{2}}}$, α = 0.05, $U_{\alpha /2}$= 1.96; the allowable error δ was taken as 0.1 [29]. According to the presurvey, the standard deviations of the dimensions of knowledge, attitude and practice were 2.43, 9.00 and 8.27, respectively, with a maximum standard deviation σ = 9.00. According to the formula, the sample size was calculated to be n ≈ 384. Considering the loss of samples, an additional 20% was added making the final sample size a total of 461 participants.

The participants were selected using convenience sampling. The inclusion criteria were as follows: ① registered nurses in tertiary hospitals; ② currently working in the adult ICU for more than 1 year; and ③relevant working experience in IMV treatment and nursing. The exclusion criteria were as follows: ① regular training and refresher nurses; ② nurses on leave for more than 1 month because of illness or maternity leave; and ③ nurses who do not directly care for patients and have been engaged in general affairs or office work for an extended period of time.

### Questionnaire

**(1) General information questionnaire.** On the basis of the literature review and study purpose, researchers freely designed the questionnaire to include basic information. This questionnaire consisted of a total of 15 questions concerning the nurses’ demographic and occupational characteristics, including the following: gender, age; the city where the hospital is located; the type of hospital, department, working years in the ICU, title, position, educational level, and form of employment; specialty nurse certificate type; whether the department has a protocolized weaning plan; whether the department has an extubation protocol for tracheal intubation patients; the department’s weaning leader; and the role of nurses in weaning decision-making. The role of nurses in weaning decision-making was measured on a 0 ~ 10 point Likert scale, with 10 representing complete autonomy and 0 representing no effect [30].
**(2) Knowledge, attitude and practice questionnaire on protocolized weaning from IMV.** The research team designed the questionnaire, which was theoretically based of the knowledge-attitude-practice (KAP) theory. KAP theory [31] is the most commonly used model to explain how personal knowledge and beliefs affect health behavior change. It classifies human behavior change into three continuous processes: acquiring knowledge, generating beliefs and forming behaviors. The questionnaire development process was as follows: under the guidance of KAP theory, 50 initial items of the questionnaire were formed through a literature review and group discussion. Seventeen critical care experts were invited to Delphi for an expert consultation, and the items of the questionnaire were modified, increased or decreased according to their expert opinions. Nine critical care experts were invited to evaluate the content validity of the items. The item-level CVI (I-CVI) of each item ranged from 0.89 ~ 1.00. In accordance with the item retention standard, of I-CVI ≥ 0.78 [32], no items were deleted. Eleven nurses of different ages, genders, professional titles and working years in the comprehensive ICU of a tertiary hospital in Chengdu were selected to participate in a pilot questionnaire test. Through the pilot test, we evaluated the clarity and response time of the content of the questionnaire and simplified the presentation of Item P9; the remaining items remained unchanged, resulting in the formation of an initial questionnaire of 49 items. From July 2023 to August 2023, 332 adult ICU nurses from 8 tertiary hospitals in Sichuan Province were presurveyed with the initial questionnaire. Item analysis, reliability analysis and exploratory factor analysis (EFA) were performed to delete items and form a final questionnaire with 39 items. The Cronbach’s α coefficient of the final questionnaire was 0.935, the half reliability coefficient was 0.729, and the Cronbach’s α coefficients of the knowledge, attitude and practice dimensions were 0.717, 0.955 and 0.936, respectively. From September 2023 to October 2023, 413 adult ICU nurses in Sichuan, Chongqing and Nanjing tertiary hospitals were surveyed with the final version of the questionnaire. To ensure that the sample source of the confirmatory factor analysis (CFA) differed from that of the EFA, the hospitals involved in the presurvey were excluded when the questionnaires were distributed in the formal survey from September to October 2023. A total of 392 valid questionnaires from the formal survey were used for CFA. The initial model results revealed that the chi square degree of freedom ratio （*χ2/df*) was 3.280, the root mean square error (RMSEA) was 0.076, and the root mean square residual error (RMR) was 0.028. The model fitting results were acceptable, and the entries were not deleted. Finally, a formal questionnaire with 39 entries was formed. The formal questionnaire included three dimensions: (1) knowledge (the concept and precautions of protocolized weaning, weaning screening and SBT evaluation, SBT methods, extubation knowledge); (2) attitude (professional cognition, personal beliefs, learning willingness); and (3) practice (implementation of weaning screening assessment and SBT, implementation of extubation). The knowledge dimension included objective questions, such as single-choice and multiple-choice questions. If the answer was correct, 1 point was assigned; if the answer was incorrect or unclear, 0 points were assigned. All items of the attitude and practice dimensions were scored on a 5-point Likert scale ranging from “strongly disagree” to “strongly agree”, with scores ranging from 1 to 5 points.

### Data collection

WENJUANXING is a professional online questionnaire survey tool in China that is used to distribute and collect questionnaires. From September 2023 to November 2023, we contacted the head nurses of relevant departments through WECHAT and asked the head nurses to distribute the WENJUANXING survey QR code and the inclusion criteria of participants to the nurses of the ICU. Nurses who met the inclusion criteria voluntarily participated in the survey after reading the guide words. In the later stage, the questionnaire data were directly exported through the back-end of WENJUANXING, and the questionnaires with a response time <180 seconds, obvious rule answers or inconsistencies were eliminated. The questionnaires were numbered and then imported into SPSS 25.0 software for statistical analysis.

### Statistical analysis

Count data are represented as the frequency and percentage; normally distributed or approximately normally distributed data are expressed as the mean ± standard deviation; nonnormally distributed data are represented as the median. Two independent sample t tests and analysis of variance were used for univariate analysis, and the statistical significance was set at *p* < 0.05. Multiple linear regression analysis was performed for the variables with significant univariate analysis and the variables with *P* < 0.1.

### Ethical considerations

The study was approved by the Ethics Committee of Sichuan Provincial People’s Hospital on April 14, 2023. Participants were informed that completion of the questionnaire was voluntary and submission of responses constituted consent to participation.

## Results

### Characteristics of the participants

A total of 700 questionnaires were collected. After eliminating the invalid questionnaires, 643 valid questionnaires remained, and the effective recovery rate of the questionnaire was 91.86%. Among the 643 respondents, 102 worked in Nanjing, 31 in Chongqing, 249 in Chengdu, and 261 in other cities in Sichuan except Chengdu; A total of 73 males and 570 females participated with an average age of 31.60 ± 5.05 years, ranging from 21 to 51 years; Most of the respondents were employed in teaching hospitals, accounting for 89.42%; General ICU (GICU) was the main department, accounting for 65.47%; The majority of ICU working years were 6 ~ 10 years, accounting for 34.21%; The professional titles of senior nurse and nurse-in-charge were in the majority, accounting for 43.08% and 42.92%, respectively; The highest education level of most respondents was Bachelor’s degree, accounting for 87.40%; The employment form was mostly contract, accounting for 70.30%; 42.92% of the respondents were intensive care specialist nurses; 86.00% of the respondents’ weaning was led by doctors; The median score of ICU nurses’ role in weaning decision was 6 (4, 8). Details of the participants are listed in Table 1.

**Table 1:** Characteristics of the study population (n = 643).

Characteristics	N (%)
Gender	
Male	73 (11.35)
Female	570 (88.65)
Age (y)	
≤25	57 (8.86)
26 ~ 30	186 (28.93)
31 ~ 40	363 (56.45)
41 ~ 50	34 (5.29)
>50	3(0.47)
Teaching hospital	
No	68 (10.58)
Yes	575 (89.42)
Types of ICU	
GICU	421 (65.47)
EICU	43 (6.69)
SICU	61 (9.49)
other ICUs	118 (18.45)
Years of ICU working experience(y)	
≤5	243 (37.79)
6 ~ 10	220 (34.21)
11 ~ 15	146 (22.71)
16 ~ 20	29 (4.51)
>20	5 (0.78)
Professional title	
Nurse	56 (8.71)
Senior nurse	277 (43.08)
Nurse-in-charge	276 (42.92)
Associate chief nurse and above	34 (5.29)
Position	
Clinical nursing teacher	182 (28.30)
Nursing team leader	100 (28.30)
Both clinical nursing teacher and nursing team leader	31 (4.82)
Head nurse	40 (4.82)
None of above	290 (45.10)
Highest education level	
Junior college or below	70 (10.89)
Bachelor’s degree	562 (87.40)
Master degree or above	11 (1.71)
Employment form	
Formal	150 (23.33)
Contract	452 (70.30)
Other	41 (70.30)
Intensive care specialist nurse	
Yes	290 (45.10)
No	353 (54.90)
Whether the ICU has a protocolized weaning program	
Not have	120 (18.66)
Yes, and strictly enforced	289 (44.95)
Yes, but not strictly enforced	234 (36.39)
Whether the ICU has a tracheal extubation procedure	
Not have	75 (11.66)
Yes, and strictly enforced	393 (61.12)
Yes, but not strictly enforced	175 (27.22)
Leader of the weaning	
RTs	25 (27.22)
Nurse	65 (10.11)
Doctor	553 (86.00)
The role of nurses in weaning decision (score)	
0 ~ 3	76 (11.82)
4 ~ 6	315 (48.99)
7 ~ 10	252 (39.19)
Whether the nurse has participated in weaning training after working	
Yes	366 (86.00)
No	277 (43.08)
The number of weaning training times in the past 3 years	
0	277 (43.08)
②1 ~ 3times	243 (37.79)
③4 ~ 6times	63 (9.80)
④ ≥ 7times	60 (9.33)
Whether the nurse is willing to participate in the protocolized weaning training	
Yes	608 (94.56)
No	35 (5.44)

Abbreviations: GICU, General Intensive Care Unit; EICU, Emergency Intensive Care Unit; SICU, Surgical Intensive Care Unit.

### Experience and demand status of protocolized weaning training

**Fig 1:**
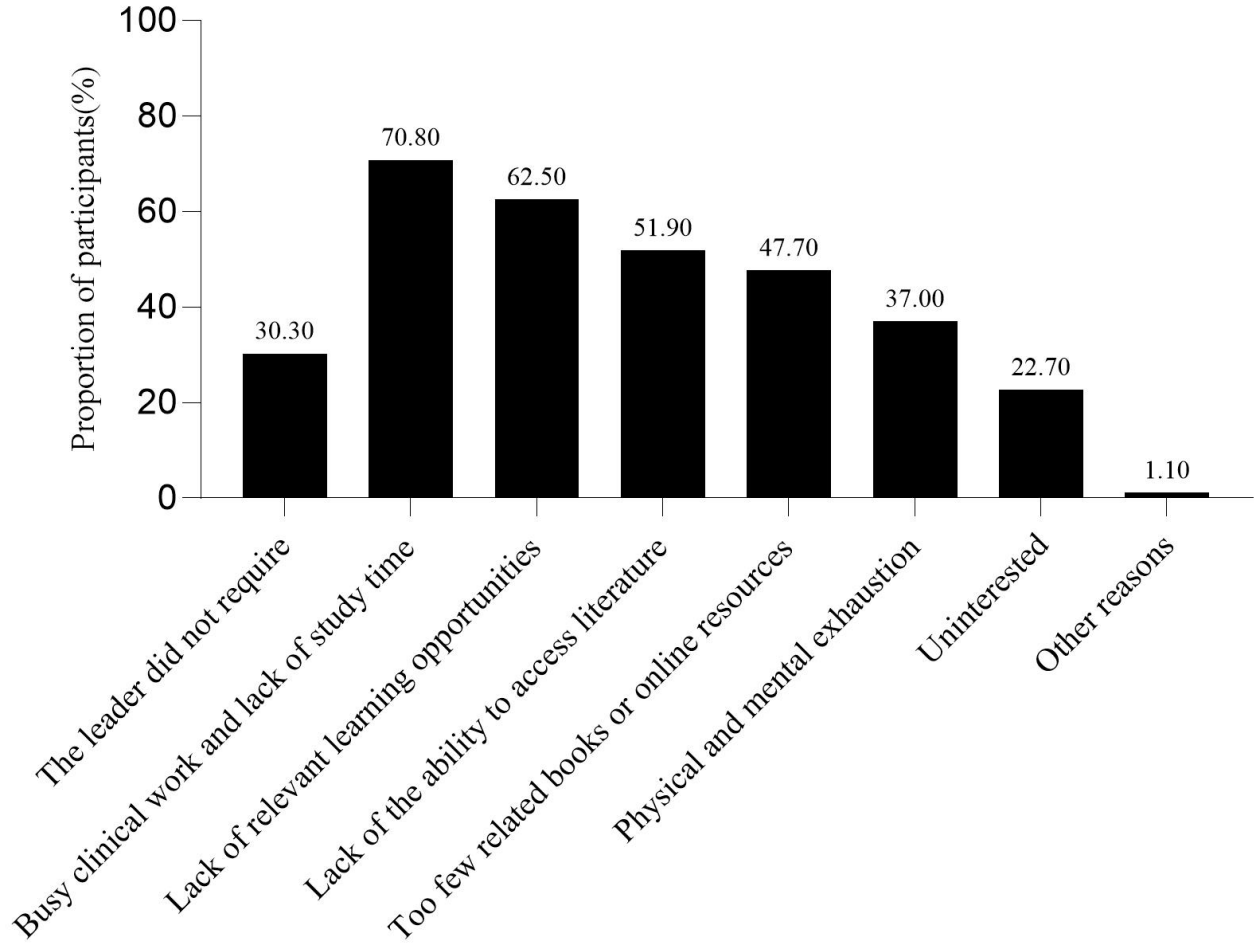
Reasons affecting ICU nurses’ learning of weaning-related knowledge.

**Fig 2:**
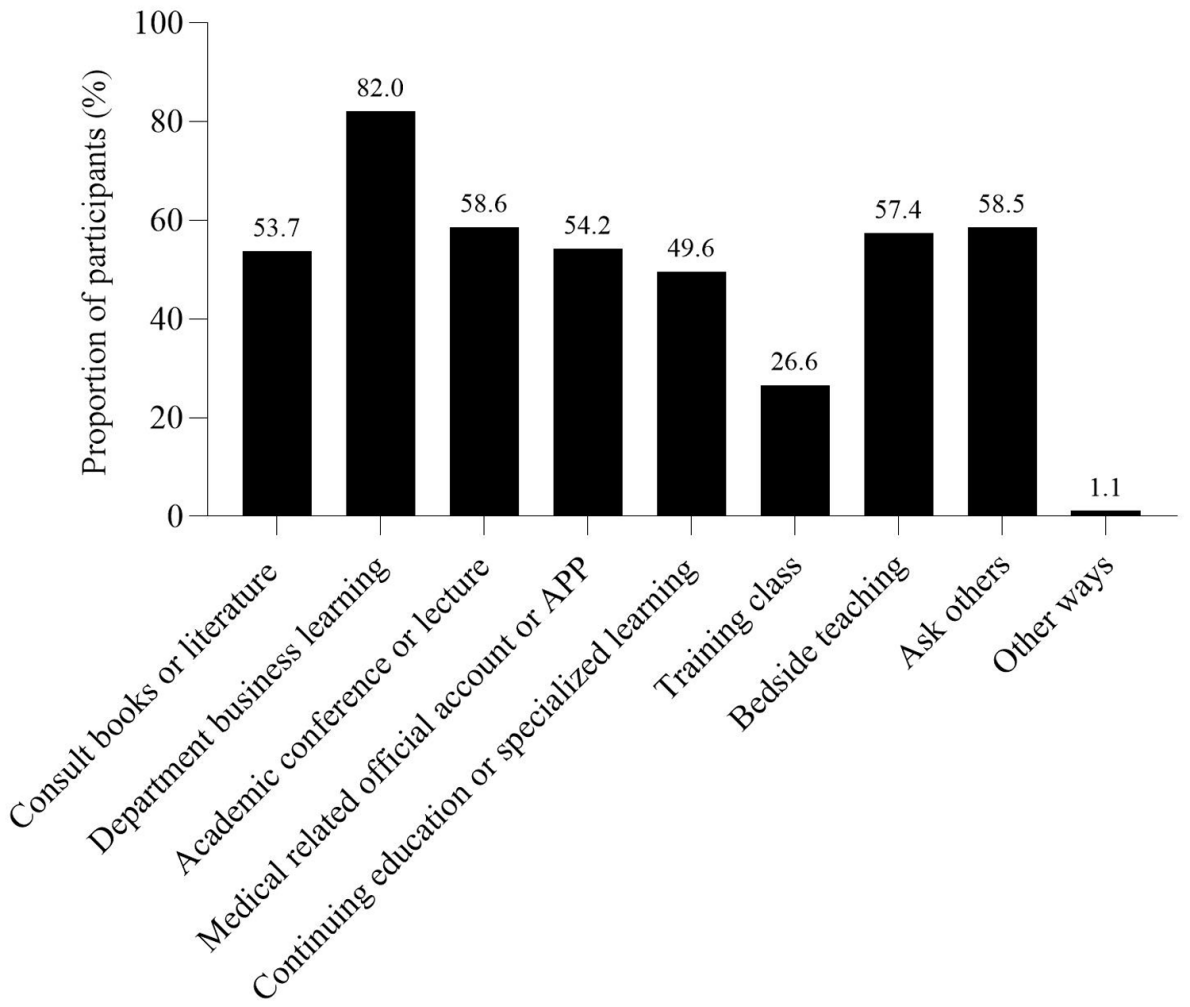
Methods of ICU nurses’ learning weaning related knowledge after work.

**Fig 3:**
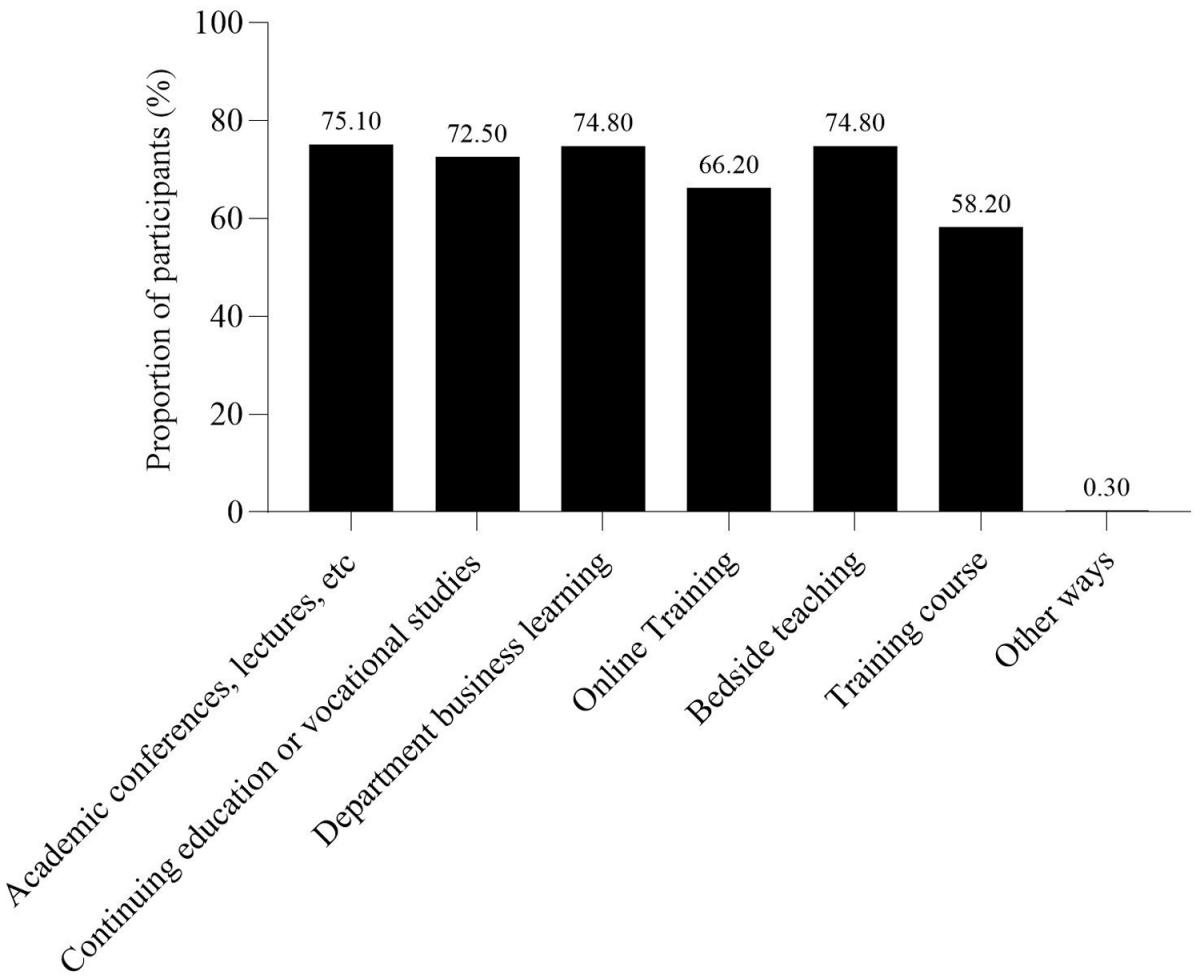
Ways for ICU nurses wishing to attend protocolized weaning training.

**Fig 4:**
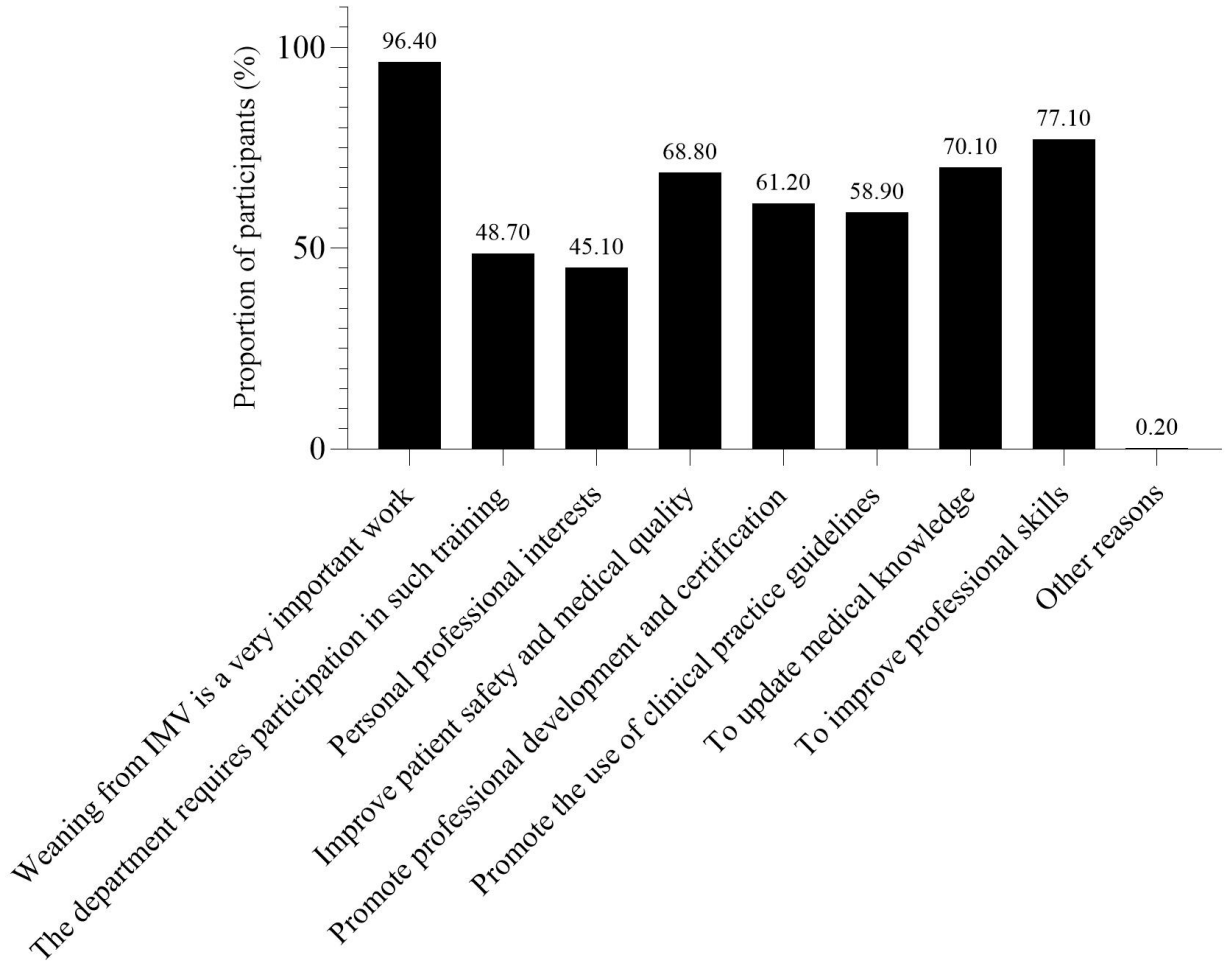
Reasons for ICU nurses’ willingness to participate in the training of protocolized weaning.

The survey results showed that 43.08% of nurses did not participate in the training of weaning-related knowledge after work. The respondents believed that the primary reason affecting their learning of weaning-related knowledge was the busy clinical work and lack of learning time, followed by the lack of relevant learning opportunities, and the lack of ability to access literature (Fig 1). This is similar to the study conducted by Jansson, M. et al.[33]. The study by Jansson, M. et al. indicated that the main factors hindering ICU nurses from implementing evidence-based guidelines for preventing VAP were inadequate resources and disagreement with the results as well as lack of time, skills, knowledge and guidance. The first five ways to learn about weaning after work were: department business learning, academic conferences or lectures, ask others, bedside teaching, and medical related official account or app (Fig 2). Among the respondents, 94.56% of the nurses expressed their willingness to participate in the knowledge training related to protocolized weaning. The first three ways to participate in the knowledge training of weaning were academic conferences, lectures, etc., bedside teaching, and department business learning (Fig 3). The first three reasons for being willing to participate in the training on protocolized weaning-related knowledge were as follows: weaning from IMV is a very important work, to improve professional skills, and to update medical knowledge (Fig 4).

### KAP Scores of protocolized weaning

The survey showed that the total score of ICU nurses’ IMV protocolized weaning KAP questionnaire and the average score of knowledge, attitude and practice were (118.27 ± 15.29), (10.21 ± 2.30), (65.97 ± 8.07) and (42.09 ± 8.53), respectively, as presented in Table 2.

**Table 2:** KAP Scores of ICU nurses on protocolized weaning of IMV (n = 643).

Items	Scoring range	Score($\overset{―}{x}$±s)	Standard score($\overset{―}{x}$±s)
Knowledge dimension	2 ~ 13	10.21 ± 2.30	78.50 ± 16.67
Attitude dimension	21 ~ 75	65.97 ± 8.07	87.96 ± 10.76
Practice dimension	11 ~ 55	42.09 ± 8.53	76.53 ± 15.51
Total questionnaire	48 ~ 143	118.27 ± 15.29	82.70 ± 10.69

The standardized scores of the questionnaire and each dimension were calculated according to the percentage system. The calculation formula is [34]: standard score = average score/total score × 100%. According to the standard score, the grade is divided. The standard score ≥ 85 points is considered an excellent level, 60 points ≤ standard score < 85 points is considered a moderate level, and the standard score < 60 points is considered a poor level. The results showed that the total score of knowledge, attitude and practice of ICU nurses’ IMV protocolized weaning was (82.70 ± 10.69), and only 41.84% of ICU nurses were at an excellent level. The standard score of the knowledge dimension was (78.50 ± 16.67), 56.92% of ICU nurses’ knowledge score was at the moderate level, and only 32.35% of nurses were at the excellent level; The standard score of the attitude dimension was (87.96 ± 10.76), and 56.45% of ICU nurses were at an excellent level; The standard score of the practice dimension was (76.53 ± 15.51), and 52.88% of ICU nurses were at the moderate level, and only 29.70% of nurses were at an excellent level. The knowledge and practice of ICU nurses on protocolized weaning of IMV were at a moderate level, and their attitude was at an excellent level, as presented in Table 3.

**Table 3:** Standard scores of KAP of protocolized weaning of IMV in ICU nurses (n = 643).

Class of grades	Knowledge score	Attitude score	Practice score	Total questionnaire
n(%)	n(%)	n(%)	n(%)
Excellent	208(32.35)	363(56.45)	191(29.70)	269(41.84)
moderate	366(56.92)	272(42.30)	340(52.88)	362(56.30)
Poor	69(10.73)	8(1.24)	112(17.42)	12(1.87

### Correlation analysis of KAP scores of protocolized weaning

Pearson correlation analysis of ICU nurses’ IMV protocolized weaning knowledge, attitude and practice dimension scores (Table 4) showed that a positive correlation among them (*p* < 0.01).

**Table 4:** Correlation analysis of KAP scores of IMV protocolized weaning among ICU nurses (n = 643).

Dimension	Knowledge score	Attitude score	Practice score
Knowledge score	1		
Attitude score	.274**	1	
Practice score	.325**	.491**	1.00

Note: ** indicates significant correlation at 0.01 level (two-tailed).

### Single factor analysis of influencing KAP of protocolized weaning

(1) Significant differences were observed in the scores of nurses’ ICU type, professional title, position, employment form, whether the ICU had a protocolized weaning program, whether the ICU had a tracheal extubation procedure, whether they had participated in weaning training after work, the number of weaning training times in the past 3 years, and whether they were willing to participate in the protocolized weaning training (p < 0.05), as delineated in Table 5.

**Table 5:** Univariate analysis of influencing factors on the knowledge dimension score of ICU nurses toward IMV protocolized weaning (n = 643).

Characteristics	Group	n	Knowledge score ($\overset{―}{x}$*±s*)	*t*/*F*	*P*	LSD pairwise comparison
Gender	Male	73	10.32 ± 2.34	0.433	0.665	——
	Female	570	10.19 ± 2.29			
Age（y）	≤25	57	9.81 ± 2.22	1.727	0.142	——
	26 ~ 30	186	10.02 ± 2.34			
	31 ~ 40	363	10.39 ± 2.25			
	41 ~ 50	34	9.82 ± 2.59			
	＞50	3	11.33 ± 0.58			
Teaching hospital	Yes	575	10.26 ± 2.24	1.731	0.084	——
	No	68	9.75 ± 2.74			
Types of ICU	①GICU	421	10.22 ± 2.22	5.754	0.001	①③* ①④* ②④* ③④*
	②SICU	61	10.52 ± 2.55			
	③EICU	43	11.21 ± 1.68			
	④Other ICUs	118	9.63 ± 2.49			
Years of ICU working experience(y)	① ≤ 5	243	9.99 ± 2.24	1.928	0.104	①⑤*
	②6 ~ 10	220	10.25 ± 2.32			
	③11 ~ 15	146	10.45 ± 2.42			
	④16 ~ 20	29	10.07 ± 1.93			
	⑤ ﹥ 20	5	12.2 ± 0.84			
Professional title	①Nurse	56	9.79 ± 2.51	5.151	0.002	①③* ①④* ②③* ②④*
	②Senior nurse	277	9.91 ± 2.39			
	③Nurse-in-charge	276	10.49 ± 2.19			
	④Associate chief nurse and above	34	11.03 ± 1.45			
Position	①Clinical nursing teacher	182	10.47 ± 2.16	5.192	<0.001	①⑤* ②⑤* ③⑤*
	②Nursing team leader	100	10.58 ± 2.15			
	③Both clinical nursing teacher and nursing team leader	31	11.13 ± 1.41			
	④Head nurse	40	10.4 ± 2.41			
	⑤None of above	290	9.78 ± 2.42			
Highest education level	Junior college or below	70	10.03 ± 2.07	0.340	0.712	——
	Bachelor’s degree	562	10.22 ± 2.34			
	Master degree or above	11	10.55 ± 1.37			
Employment form	①Formal	150	10.69 ± 2.26	4.807	0.008	①②* ①③* ②③*
	②Contract	452	10.09 ± 2.28			
	③Other	41	9.73 ± 2.43			
Intensive care specialist nurse	Yes	290	10.37 ± 2.16	1.640	0.101	——
	No	353	10.07 ± 2.40			
Whether the ICU has a protocolized weaning program	①Not have	120	9.01 ± 2.99	22.339	<0.001	①②* ①③*
	②Yes, but not strictly enforced	234	10.33 ± 2.16			
	③Yes, and strictly enforced	289	10.6 ± 1.87			
Whether the ICU has a tracheal extubation procedure	①Not have	75	9.13 ± 3.27	12.990	<0.001	①②* ①③* ②③*
	②Yes, but not strictly enforced	175	9.98 ± 2.39			
	③Yes, and strictly enforced	393	10.51 ± 1.94			
Leader of the weaning	Doctor	553	10.21 ± 2.32	0.220	0.803	——
	Nurse	65	10.06 ± 2.10			
	RTs	25	10.40 ± 2.26			
The role of nurses in weaning decision-making (score)	0 ~ 3	76	9.75 ± 2.69	1.764	0.172	——
	4 ~ 6	315	10.23 ± 2.26			
	7 ~ 10	252	10.31 ± 2.21			
Whether the nurse has participated in weaning training after working	Yes	366	10.67 ± 1.95	5.864	<0.001	——
	No	277	9.59 ± 2.57			
The number of weaning training times in the past 3 years	①0	277	9.59 ± 2.57	13.420	<0.001	①②* ①③* ①④*
	②1 ~ 3times	243	10.60 ± 1.98			
	③4–6times	63	10.51 ± 2.03			
	④ ≥ 7times	60	11.12 ± 1.67			
Whether the nurse is willing to participate in the protocolized weaning training	Yes	608	10.28 ± 2.20	2.508	0.017	——
	No	35	8.86 ± 3.32			

Note: The outcomes are unadjusted, * represents *P* < 0.05.

Abbreviations: GICU, General Intensive Care Unit; EICU, Emergency Intensive Care Unit; SICU, Surgical Intensive Care Unit.

(2) Significant differences were observed in the scores of ICU nurses’ IMV protocolized weaning attitudes in terms of ICU type, professional title, whether the ICU had a protocolized weaning program, whether the ICU had a tracheal extubation procedure, the role of nurses in weaning decision-making, whether they had participated in weaning training after work, the number of weaning trainings in the past 3 years, and whether they were willing to participate in the training of protocolized weaning (p < 0.05), as illustrated in Table 6.

**Table 6:** Univariate analysis of influencing factors on the attitude dimension score of ICU nurses toward IMV protocolized weaning (n = 643).

Characteristics	Group	n	Attitude score ($\overset{―}{x}$*±s*)	*t*/*F*	*P*	LSD pairwise comparison
Gender	Male	73	65.96 ± 10.86	0.010	0.992	——
	Female	570	65.97 ± 7.66			
Age（y）	≤25	57	64.28 ± 8.18	1.141	0.336	——
	26 ~ 30	186	65.61 ± 8.46			
	31 ~ 40	363	66.46 ± 7.94			
	41 ~ 50	34	65.74 ± 6.69			
	＞50	3	63.33 ± 10.41			
Teaching hospital	Yes	575	66.03 ± 7.96	0.587	0.557	——
	No	68	65.43 ± 9.04			
Types of ICU	①GICU	421	65.21 ± 7.87	3.947	0.008	①②* ①③* ③④*
	②SICU	61	67.70 ± 7.74			
	③EICU	43	68.23 ± 7.17			
	④Other ICUs	118	66.96 ± 8.91			
Years of ICU working experience (y)	① ≤ 5	243	65.73 ± 8.31	1.286	0.274	②③*
	②6 ~ 10	220	65.52 ± 8.54			
	③11 ~ 15	146	67.27 ± 7.15			
	④16 ~ 20	29	65.07 ± 6.14			
	⑤ ﹥ 20	5	64.80 ± 9.09			
Professional title	①Nurse	56	62.71 ± 10.94	4.204	0.006	①②* ①③* ①④*
	②Senior nurse	277	66.16 ± 8.13			
	③Nurse-in-charge	276	66.15 ± 7.28			
	④Associate chief nurse and above	34	68.38 ± 7.08			
Position	①Clinical nursing teacher	182	66.24 ± 7.39	1.459	0.21	④⑤*
	②Nursing team leader	100	66.39 ± 7.46			
	③Both clinical nursing teacher and nursing team leader	31	66.90 ± 6.99			
	④Head nurse	40	68.08 ± 7.16			
	⑤None of above	290	65.27 ± 8.84			
Highest education level	Junior college or below	70	65.84 ± 8.60	0.016	0.985	——
	Bachelor’s degree	562	65.99 ± 8.03			
	Master degree or above	11	65.73 ± 7.38			
Employment form	Formal	150	66.33 ± 8.82	1.478	0.229	——
	Contract	452	66.04 ± 8.24			
	Other	41	63.93 ± 6.93			
Intensive care specialist nurse	Yes	276	66.14 ± 7.83	0.496	0.620	——
	No	353	65.83 ± 8.27			
Whether the ICU has a protocolized weaning program	①Not have	120	63.61 ± 8.17	12.482	<0.001	①③* ②③*
	②Yes, but not strictly enforced	234	65.18 ± 8.03			
	③Yes, and strictly enforced	289	67.59 ± 7.75			
Whether the ICU has a tracheal extubation procedure	①Not have	75	63.84 ± 8.32	7.352	0.001	①③* ②③*
	②Yes, but not strictly enforced	175	64.77 ± 8.38			
	③Yes, and strictly enforced	393	66.91 ± 7.75			
Leader of the weaning	Doctor	553	65.94 ± 7.90	0.032	0.968	——
	Nurse	65	66.08 ± 9.55			
	RTs	25	66.32 ± 8.00			
The role of nurses in weaning decision-making (score)	①0 ~ 3	76	62.49 ± 7.74	20.821	<0.001	①②* ①③* ②③*
	②4 ~ 6	315	64.98 ± 7.80			
	③7 ~ 10	252	68.26 ± 7.91			
Whether the nurse has participated in weaning training after working	Yes	366	66.88 ± 8.06	3.309	0.001	——
	No	277	64.77 ± 7.94			
The number of weaning training times in the past 3 years	①0	277	64.77 ± 7.94	6.168	<0.001	①③* ①④* ②③*
	②1-3	242	66.14 ± 7.67			
	③4-6	63	68.59 ± 7.76			
	④ ≥ 7	60	68.33 ± 9.32			
Whether the nurse is willing to participate in the protocolized weaning training	Yes	607	66.26 ± 7.92	3.912	<0.001	——
	No	35	60.83 ± 9.15			

Note: The outcomes are unadjusted, * represents *P* < 0.05.

Abbreviations: GICU, General Intensive Care Unit; EICU, Emergency Intensive Care Unit; SICU, Surgical Intensive Care Unit.

(3) Significant differences were observed in the scores of ICU nurses’ IMV protocolized weaning practice among factors such as gender, types of ICU, working years in ICU, whether the ICU had a protocolized weaning program, whether the ICU had a tracheal extubation procedure, the role of nurses in weaning decision-making, whether they have participated in weaning training after work, the number of weaning training times in the past 3 years, and whether they were willing to participate in the training of protocolized weaning (*p* < 0.05), as indicated in Table 7.

**Table 7:** Univariate analysis of influencing factors on the practice dimension score of ICU nurses toward IMV protocolized weaning(n = 643).

Characteristics	Group	n	Practice score ($\overset{―}{x}$*±s*)	*t*/*F*	*P*	LSD pairwise comparison
Gender	Male	73	44.70 ± 8.95	2.788	0.005	——
	Female	570	41.76 ± 8.42			
Age(y)	≤25	57	40.70 ± 8.56	0.82	0.51	——
	26 ~ 30	186	41.66 ± 8.73			
	31 ~ 40	363	42.58 ± 8.36			
	41 ~ 50	34	41.62 ± 9.40			
	＞50	3	42.33 ± 3.79			
Teaching hospital	Yes	575	42.28 ± 8.48	1.660	0.097	——
	No	68	40.47 ± 8.85			
Types of ICU	①GICU	421	41.41 ± 8.14	9.891	<0.001	①②* ②③* ②④*
	②SICU	61	47.30 ± 5.97			
	③EICU	43	43.84 ± 8.74			
	④Other ICUs	118	41.20 ± 9.88			
Years of ICU working experience(y)	① ≤ 5	243	41.27 ± 8.82	1.700	0.148	①③*
	②6 ~ 10	220	42.12 ± 8.88			
	③11 ~ 15	146	43.49 ± 7.67			
	④16 ~ 20	29	42.17 ± 6.85			
	⑤ ﹥ 20	5	39.20 ± 7.73			
Professional title	Nurse	56	40.48 ± 8.55	1.430	0.233	——
	Senior nurse	277	42.35 ± 8.48			
	Nurse-in-charge	276	41.91 ± 8.63			
	Associate chief nurse and above	34	44.12 ± 7.88			
Position	①Clinical nursing teacher	182	42.19 ± 7.97	1.993	0.094	③⑤*
	②Nursing team leader	100	43.05 ± 8.47			
	③Both clinical nursing teacher and nursing team leader	31	44.81 ± 7.30			
	④Head nurse	40	43.23 ± 8.33			
	⑤None of above	290	41.26 ± 8.97			
Highest education level	Junior college or below	70	42.83 ± 9.18	1.585	0.206	——
	Bachelor’s degree	562	41.92 ± 8.46			
	Master degree or above	11	46.09 ± 7.27			
Employment form	Formal	150	42.61 ± 8.25	0.732	0.481	——
	Contract	452	42.04 ± 8.65			
	Other	41	40.83 ± 8.22			
Intensive care specialist nurse	Yes	290	42.62 ± 7.86	1.455	0.146	——
	No	353	41.65 ± 9.03			
Whether the ICU has a protocolized weaning program	①Not have	120	38.22 ± 8.19	25.825	<0.001	①②* ①③* ②③*
	②Yes, but not strictly enforced	234	41.25 ± 7.91			
	③Yes, and strictly enforced	289	44.38 ± 8.46			
Whether the ICU has a tracheal extubation procedure	①Not have	75	38.61 ± 9.15	15.070	<0.001	①③* ②③*
	②Yes, but not strictly enforced	175	40.49 ± 7.95			
	③Yes, and strictly enforced	393	43.47 ± 8.36			
Leader of the weaning	Doctor	553	42.02 ± 8.41	0.175	0.839	——
	Nurse	65	42.68 ± 9.27			
	RTs	25	42.20 ± 9.48			
The role of nurses in weaning decision-making (score)	①0 ~ 3	76	37.16 ± 8.38	23.341	<0.001	①②* ①③* ②③*
	②4 ~ 6	315	41.52 ± 8.24			
	③7 ~ 10	252	44.29 ± 8.21			
Whether the nurse has participated in weaning training after working	Yes	366	44.33 ± 7.78	8.009	<0.001	——
	No	277	39.14 ± 8.59			
The number of weaning training times in the past 3 years	0	277	39.14 ± 8.59	29.247	<0.001	——
	1-3 times	242	43.14 ± 7.57			
	4-6 times	63	45.14 ± 7.40			
		≥7 times	60	48.38 ± 7.66		
Whether the nurse is willing to participate in the protocolized weaning training	Yes	608	42.36 ± 8.43	3.352	0.001	——
	No	35	37.43 ± 8.97			

Note: The outcomes are unadjusted, * represents *P* < 0.05.

Abbreviations: GICU, General Intensive Care Unit; EICU, Emergency Intensive Care Unit; SICU, Surgical Intensive Care Unit.

### Multiple linear regression analysis of influencing KAP of protocolized weaning

Taking the scores of each dimension of ICU nurses’ IMV protocolized weaning knowledge, attitude and practice were considered as the dependent variable (y), and the statistically significant factors (*p* < 0.05) in the univariate analysis were considered as the independent variable (x). To avoid the omission of important influencing factors, the variables with *p* < 0.1 in the univariate analysis were also included [35], and multiple linear regression analysis was conducted one by one.

Multiple linear regression analysis (Table 8) showed that five factors, including the employment form, whether the ICU has a protocolized weaning program, whether the nurse is willing to participate in the protocolized weaning training, the ICU type, and the position were the main factors influencing the score of ICU nurses’ IMV protocolized weaning knowledge, which could explain the variation of 14.3% of the total knowledge score. There were also five factors that influence the attitude score, and they were as follows: the professional title, the ICU type, whether the ICU has a protocolized weaning program, the role of nurses in weaning decision-making, and whether the nurse is willing to participate in the protocolized weaning training. The five factors could explain 12.3% of the total variation of the total score of attitudes. In addition, gender, ICU types, the position, whether the ICU has a protocolized weaning program, the role of nurses in weaning decision-making, the number of weaning training times in the past 3 years, and whether the nurse is willing to participate in the protocolized weaning training, were the main factors influencing the score of ICU nurses’ IMV protocolized weaning practice, and the seven factors could explain 23.4% of the total variation.

**Table 8:** Multiple linear regression analysis of ICU nurses’ KAP about IMV protocolized weaning.

Variables	*B*	SE	*β*	*t*	*P*	95%CI	
						Lower limit	Upper limit
**Knowledge**							
Constants	5.291	0.691		7.660	< 0.001	3.935	6.648
Employment form	0.376	0.181	0.085	2.069	0.039	0.019	0.732
Whether your ICU has a protocolized weaning program	0.516	0.148	0.169	3.480	0.001	0.225	0.808
Whether you are willing to participate in the protocolized weaning training	1.024	0.377	0.101	2.717	0.007	0.284	1.763
ICU type							
ICU	Ref						
Other ICU	−0.470	0.226	−0.079	−2.077	0.038	−0.914	−0.026
EICU	0.765	0.346	0.083	2.212	0.027	0.086	1.444
Position							
No position	Ref						
Clinical nursing teacher	0.671	0.225	0.132	2.985	0.003	0.229	1.112
Nursing team leader	0.579	0.268	0.091	2.162	0.031	0.053	1.106
Both clinical nursing teacher and nursing team leader	0.932	0.431	0.087	2.165	0.031	0.087	1.778
**Attitude**							
Constants	47.956	2.192		21.873	< 0.001	43.650	52.261
ICU type							
ICU	Ref						
SICU	2.295	1.049	0.083	2.187	0.029	0.234	4.355
Other ICU	2.081	0.794	0.100	2.621	0.009	0.522	3.640
EICU	2.543	1.216	0.079	2.092	0.037	0.156	4.931
Professional title	1.416	0.430	0.127	3.295	0.001	0.572	2.260
Whether your ICU has a protocolized weaning program	1.447	0.529	0.135	2.735	0.006	0.408	2.485
The role of nurses in weaning decision-making	2.342	0.467	0.192	5.014	< 0.001	1.425	3.259
Whether you are willing to participate in the protocolized weaning training	4.438	1.333	0.125	3.328	0.001	1.819	7.056
**Practice**							
Constants	20.919	2.436		8.589	< 0.001	16.137	25.702
Gender	2.878	0.954	0.107	3.018	0.003	1.006	4.751
ICU type							
SICU	5.444	1.039	0.187	5.240	< 0.001	3.404	7.484
Whether your ICU has a protocolized weaning program	1.599	0.526	0.141	3.038	0.002	0.565	2.632
The role of nurses in weaning decision making	2.175	0.462	0.168	4.707	< 0.001	1.267	3.082
The number of weaning training times in the past 3 years	2.201	0.522	0.242	4.219	< 0.001	1.176	3.225
Whether you are willing to participate in the protocolized weaning training	3.195	1.325	0.085	2.411	0.016	0.593	5.797
Position							
No position	Ref						
Nursing supervisor	3.390	1.312	0.096	2.583	0.010	0.813	5.967
Clinical nursing teacher	1.558	0.757	0.082	2.059	0.040	0.072	3.044

Note: Knowledge Dimension Model: R^2^ = 0.163, adjusted R^2^ = 0.143; F = 8.141, p < 0.001.

Attitude Dimension Model: R^2^ = 0.136, adjusted R^2^ = 0.123; F = 9.951, p < 0.001.

Practice Dimension Model: R^2^ = 0.253, adjusted R^2^ = 0.234; F = 13.23, p < 0.001.

Abbreviations: EICU, Emergency Intensive Care Unit, SICU, Surgical Intensive Care Unit.

Explanation of independent variable assignment: Sex: female = 1, male = 2; Teaching hospital: yes = 1, no = 0; ICU type: set dummy variable (take “general ICU” as the reference); Professional title: nurse = 1, senior nurse = 2, nurse-in-charge = 3, associate chief nurse and above=4; Position: set dummy variable (take “no position” as reference); Employment form: Other = 1, off-staff = 2, in-staff = 3; Intensive care specialist nurse: yes = 1, no = 0; Whether the ICU has a protocolized weaning program: none = 1, (yes, but not strictly enforced) =2, (yes, and strictly enforced) =3; Whether the ICU has a tracheal extubation procedure: none = 1, (yes, but not strictly enforced) =2, (yes, and strictly enforced) =3; The role of nurses in weaning decision-making (score): 0 ~ 3 points = 1, 4 ~ 6 points = 2, 7 ~ 10 points = 3; Whether the nurse has participated in weaning training after working: yes = 1, no = 0; The number of weaning training times in the past 3 years: 0 times=0, 1–3 times=1, 4–6 times=2, 7 times and above=3; Whether the nurse is willing to participate in the protocolized weaning training: yes = 1, no = 0.

## Discussion

### The KAP level of ICU nurses’ protocolized weaning needs to be improved

The results of this study showed that the standard scores of ICU nurses’ KAP of IMV protocolized weaning were (78.50 ± 16.67), (87.96 ± 10.76) and (76.53 ± 15.51), respectively. The overall level of KAP needs to be further improved, especially in the aspects of knowledge and practice. Although all these ICU nurses were from tertiary hospitals, which usually offer better education and training, their scores for knowledge and attitude were only at a moderate level. This may be related to the fact that the implementation of ventilator weaning in China was mainly led by doctors (86% of the respondents worked in departments where the weaning was led by doctors), and nurses rarely participated in ventilator weaning training after work (this study showed that only 56.92% of nurses participated in ventilator weaning training after work). Research shows continuing education can improve nurses’ professional knowledge and clinical critical thinking, promote best practices and improve patient outcomes [36]. Therefore, tertiary hospitals should enhance the training of nurses on the knowledge of protocolized weaning from mechanical ventilation to improve their knowledge level. Pearson correlation analysis of this study showed a pairwise positive correlation among ICU nurses’ KAP of IMV protocolized weaning. So once the level of knowledge improves, the levels of attitude and practice will also increase accordingly.

### Analysis on influencing factors of KAP level of protocolized weaning

**(1) Types of ICU**

The type of ICU is the factor influencing ICU nurses’ IMV protocolized weaning KAP scores. Compared with nurses in the general ICU, nurses in the emergency ICU scored higher on the knowledge level of protocolized weaning, while nurses in other ICUs scored lower on the knowledge level. The reason for this finding may be because different departments have different levels of personnel, medical cooperation, workload, weaning roles and responsibilities, or the implementation of protocolized weaning programs in each ICU was different [37]. The relationship of mutual trust and cooperation between doctors and nurses and the support from senior leaders can promote the decision of nurses to implement protocolized weaning [27]. However, the increase in workload and the decrease in nursing continuity caused by frequent shifts may cause nurses to delay the implementation of protocolized weaning procedures [38]. The values, preferences, knowledge and skills of doctors in different departments may also affect the adoption and implementation of the protocolized weaning program [37]. Therefore, a good relationship of trust and cooperation should be established between doctors and nurses. Each department should formulate a protocolized weaning program according to its own situation, clarify the role and responsibility of weaning, and promote the standardized implementation of protocolized weaning.

**(2) Professional title, position, employment form**

This study showed that nurses with higher professional titles had higher scores of attitudes toward protocolized weaning. Generally, the level of professional title reflects the degree of accumulation of clinical work experience to a certain extent [39]. The higher the professional title, the richer the work experience, and the more experienced nurses have the ability to make weaning decisions than novice nurse [40]. This is similar to the investigation conducted by Jansson, M. et al. [33] in Finland regarding the understanding of evidence-based guidelines for preventing ventilator-associated pneumonia by ICU nurses. Nurses with more experience scored higher in terms of knowledge than those with less experience. The knowledge level of protocolized weaning of teachers and nursing team leaders was higher than that of ordinary nurses, but the head nurse was not the influencing factor of knowledge level. It may be because the teachers and nursing team leaders were the people who perform well in usual work, and they were also the backbone of the department with relatively solid professional knowledge. The head nurses were mainly responsible for administrative management, less involved in clinical practice of weaning, resulting in a slightly poor mastery of related knowledge. The knowledge level of formal nurses was higher than that of contract nurses and other forms of employment, which is similar to the research results of Linghu Changlian [39]. The reason for this finding may be because formal nurses have better salary, welfare, pension insurance and other aspects than other nurses, and they are more practical and can stimulate their enthusiasm for actively learning professional knowledge. Therefore, it is suggested that the hospital should increase the number of formal nurses, stabilize the nurse team, and improve the learning enthusiasm of nurses.

**(3) Whether the ICU has a protocolized weaning program, whether the nurse is willing to participate in the training related to protocolized weaning**

These factors influence ICU nurses’ IMV protocolized weaning knowledge score, attitude score and practice score. The application of a protocolized weaning plan can standardize the weaning process, provide evidence for the operation process, and increase the confidence of nurses [37], especially for young and inexperienced ICU nurses [41]. Nurses constantly apply and implement the plan in their work. One operation is equivalent to reviewing knowledge and deepening the mastery of knowledge. The protocolized weaning plan can provide guidance [41] and auxiliary decision-making for nurses to increase their autonomy, without consulting doctors at any time, and improve the enthusiasm of nurses to participate in weaning. Therefore, it is suggested that each ICU should develop a standardized weaning plan suitable for their own department based on evidence-based and guideline recommendations, in order to enhance the enthusiasm of nurses in the weaning process. Nurses’ attitude and interest in weaning will also affect their decision concerning weaning [42]. Nurses who are willing to participate in protocolized weaning knowledge training are interested in weaning and are more willing to take the initiative to learn relevant knowledge. This suggested that managers can actively cultivate nurses who are interested in weaning from the ventilator to become specialized nurses in mechanical ventilation, and better lead the implementation of the protocolized weaning process.

**(4) The role of nurses in weaning decision-making**

This factor influences the score of ICU nurses’ IMV protocolized weaning attitude and practice. In this study, the median of nurses’ perceived autonomy in weaning decision-making was 6 (4, 8), which was higher than that of European pediatric ICU nurses’ perception of nursing autonomy in weaning decision-making of mechanical ventilation 4 (2, 6) [30]. This may be because the survey object of this study is adult ICU nurses, the risk of weaning in adults is less than that of pediatric patients, and the nurses’ perception of decision-making power is relatively higher.

Although the weaning decision is jointly made by doctors and nurses, the unclear role and responsibility of nurses in weaning can weaken the enthusiasm of nurses to participate in weaning [38]. Due to the influence of the nature of work and traditional concepts, nurses cannot jump out of the fixed thinking that “they are only the executor of medical orders”, so they feel less autonomy in weaning decision-making [43]. However, nurses who believed that they had a greater role in the weaning decision had higher attitude scores and higher practice scores for protocolized weaning. This might be because nurses who believe they play a more significant role in the decision-making process of weaning have a higher sense of professional identity. A positive sense of professional identity has a positive motivating effect on nurses [44], which can enhance their satisfaction and make them work more actively.

**(5) Gender, the number of weaning training times in the past 3 years**

These factors influence the score of ICU nurses’ IMV protocolized weaning practice. The practice score of male nurses in implementing protocolized weaning was higher than that of female nurses, which was similar to the research results of Zhang Peiyao et al.[45], possibly because male nurses had higher hands-on ability and stronger ventilator operation ability than female nurses. Our study showed that the more times nurses have participated in extubation training in the past 3 years, the higher their scores in the weaning practice. In addition, the research by Jansson, M. M. et al.[46]also indicated that compared with nurses who have not received in-service education about ventilator bundle, those who have received in-service education have a higher level of knowledge. Continuing education is essential to cultivate and maintain nurses’ knowledge and skills, which may improve the treatment effect of patients [36], shorten MV time and save medical costs [26]. The forms of continuing education and training can be diversified. For example, after Nahid and others [47] adopted workshops and multimedia training, nurses’ MV weaning decision-making skills were improved. Our study showed that the first three ways for ICU nurses to participate in weaning knowledge training were attending academic conference, lectures, etc., bedside teaching, and department business learning (Fig 3), suggesting that managers can make appropriate procedural weaning training plans and training methods according to their actual situation, and continuously improve nurses’ clinical weaning knowledge and decision-making ability. The KAP theory model believes that there is a progressive relationship among knowledge, attitude and practice. Knowledge is the basis of practice change, and the belief and attitude are the driving force of practice change [31]. This study showed that there is a positive correlation between the knowledge, attitude and practice of ICU nurses regarding the protocolized weaning process (Table 2). Therefore, to enhance nurses’ protocolized weaning ability, the primary step is to improve their relevant knowledge level.

### Limitations

Although the survey tool of this study was strictly compiled in accordance with the questionnaire development procedures, the Cronbach’s α of the attitude dimension and the practice dimension was greater than 0.9, indicating that there were redundant items [48]. When applying the questionnaire in the future, appropriate item reduction can be carried out. This study used the convenient sampling method, and most of the respondents were from Sichuan hospitals, which may have certain limitations in sample representation and difference prediction. In the future, multicenter and large-sample stratified sampling surveys can be carried out to further analyze the status quo and influencing factors of KAP of nurses in different regions and different hospitals about protocolized weaning. The study was a cross-sectional questionnaire, the results of the study were mainly based on self-reported materials, which might be prone to bias and/or overestimation of real results. Future research can use behavioral observation to investigate nurses’ behavior level and qualitative interview to investigate nurses’ attitude cognition as a supplement to the questionnaire. Additionally, in the multiple linear regression analysis, the adjusted R 2 values of the knowledge dimension model and the attitude dimension model were relatively low. This indicates that other factors (not included in the regression analysis) may influence the nurses’ knowledge and attitude, such as personal experiences, individual learning styles, or hospital-specific factors, etc. Future research could incorporate a wider range of variables to provide a more comprehensive analysis.

## Conclusion

This study showed that ICU nurses in tertiary hospitals in Sichuan, Chongqing and Nanjing have a moderate level of knowledge, a positive attitude and a moderate level of practice on protocolized weaning, and there is a positive correlation between any two of the three. The ICU type, gender, position, title, the form of employment, whether the ICU had a protocolized weaning plan, the role of nurses in the weaning decision-making, the number of weaning training times in the past 3 years, and whether the nurse is willing to participate in the protocolized weaning training were the important factors affecting the KAP of ICU nurses on IMV protocolized weaning. Managers should formulate a protocolized weaning plan to guide clinical weaning according to the situation of the department. At the same time, department managers can choose the training form preferred by nurses, increase the training frequency of protocolized weaning knowledge, improve ICU nurses’ weaning knowledge and weaning role awareness, and promote the clinical implementation of protocolized weaning, to benefit patients.

### What is already known

- The working mode of nurses puts them in a favorable position to lead the weaning process.

Nurse-led protocolized weaning can reduce the duration of mechanical ventilation and does not increase complications compared with physician-led weaning.

- Qualitative research showed that the influencing factors of nurses’ implementation of protocolized weaning mainly included the environment, patient’s condition, nurses’ own condition, education and training.

### What this paper adds

- Weaning in domestic tertiary hospitals is mainly led by doctors. Nurses are more willing to participate in the implementation of protocolized weaning, but their knowledge and practice level need to be improved.
- The main factors affecting the knowledge, attitude, and practice level of ICU nurses’ IMV protocolized weaning include ICU type, position, professional title, whether the ICU has a protocolized weaning program, the role of nurses in weaning decision-making, the number of weaning training times in the past 3 years, and whether the nurse is willing to participate in the protocolized weaning training.

## Supporting information

S1 File Research data. (XLSX)
